# Age-dependent association between obstructive sleep apnea and self-reported history of fractures: a community-based study

**DOI:** 10.1186/s12889-025-25593-w

**Published:** 2025-12-23

**Authors:** Junzhi Chen, Guangliang Shan, Yaoda Hu, Huijing He, Tong Feng, Ruohan Zhou, Ping Yuan, Miaochan Lao, Qiong Ou

**Affiliations:** 1 Department of Respiratory and Critical Care Medicine, Sleep Center, Guangdong Provincial People’s Hospital, Guangdong Academy of Medical Sciences, Southern Medical University, 106 Zhongshan Second Road, Yuexiu District, Guangzhou, 510080 China; 2 Department of Epidemiology and Statistics, Institute of Basic Medical Sciences, School of Basic Medicine, Chinese Academy of Medical Sciences, Peking Union Medical College, Beijing, China

**Keywords:** Fractures, Obstructive sleep apnea, Poor sleep quality, Drinking

## Abstract

**Background:**

The relationship between obstructive sleep apnea (OSA) and self-reported history of fractures among various age groups in the general population is unclear.

**Methods:**

Participants were recruited using a multistage probability sampling method between 2021 and 2023 in Guangdong Province, China. Sleep study was performed with a wearable type-IV monitor. OSA was defined using the oxygen desaturation index (ODI) of ≥ 5 events/h. Fracture history was self-reported according to physician diagnoses. Poor sleep quality was defined as a Pittsburgh Sleep Quality Index score > 5.

**Results:**

A total of 5,519 participants were analyzed. The mean age of participants was 52.7 years. The prevalence rates of OSA and self-reported history of fractures were 44.5% and 13%, respectively. Participants were categorized into groups based on age tertiles. In T3 group (ages 59–91), OSA was significantly associated only with self-reported previous non-traumatic fractures (OR = 3.68, 95%CI:1.79–7.57). Similar results were observed in the T2 group (ages 48–58). OSA was not associated with self-reported history of fractures in the T1 group (ages 18–47). Furthermore, participants with both OSA and poor sleep quality had a higher odds of self-reported previous traumatic fractures compared to those with OSA alone in the T3 group (OR = 1.64, 95% CI:1.02–2.65). Interactions between OSA and age, as well as between OSA and alcohol consumption, regarding self-reported history of fractures were observed (*p* < 0.05).

**Conclusions:**

OSA was independently associated with self-reported previous non-traumatic fractures in the general population. The association was age-dependent and more meaningful in the elderly population.

**Supplementary Information:**

The online version contains supplementary material available at 10.1186/s12889-025-25593-w.

## Introduction

Bone fractures are a major public concern worldwide. Between 1990 and 2019, the incidence, prevalence, and years lived with disability of fractures increased by 33.4%, 70.1%, and 65.3%, respectively [1]. Fractures can lead to reduced quality of life, increased mortality, disability, impaired psychosocial functioning, and high healthcare costs. These consequences not only create an additional burden on healthcare systems but also negatively impact individual well-being [2, 3]. Preventing fractures has become a significant global challenge.

Obstructive sleep apnea (OSA) is a prevalent chronic disease worldwide [4]. It is characterized by intermittent hypoxia during sleep due to repetitive episodes of partial or complete upper airway obstruction, leading to daytime sleepiness [5] and an increased risk of cardiovascular morbidity and mortality [6]. Furthermore, chronic OSA-related intermittent hypoxia can induce inflammation and create an acidic microenvironment in the bone, negatively affecting bone metabolism and microarchitecture [7]. This condition increases the risk of osteoporosis and osteoporosis-related fractures [8–10].

To our knowledge, no prior studies have reported an association between OSA and self-reported history of fractures in the general population. Although some studies have assessed the relationship between OSA, snoring, or nocturnal hypoxia and fractures, they primarily focused on fracture outcomes in specific sites or types [11–13]. Furthermore, none of these studies attempted to draw a representative OSA sample from the population. OSA participants in previous studies were derived from sampling the population at risk for OSA [11–13], the association between OSA and self-reported history of fractures in the general population remains unknown. More importantly, both the prevalence of OSA and fractures increase with age. Understanding the association between fractures and OSA across various age groups may help identify specific populations for targeted intervention and prevention.

Therefore, we aimed to evaluate the association between OSA and self-reported history of fractures in a large-scale general population. We hypothesized that age may modify this association.

## Materials and methods

### Study design and participants

Participants were enrolled from the Diverse Life-Course Cohort (DLCC) [14]. The DLCC was established in 2017 by the Institute of Basic Medical Sciences, the Chinese Academy of Medical Sciences. It aims to describe the etiology of specific non-communicable diseases across different populations throughout their entire life course. Details of the design, objectives, and survey methods of the DLCC have been previously published [14]. Under the consideration of project management and data sharing, the data of DLCC have been linked to the National Population Health Data Center (https://www.ncmi.cn/index.html). On which researchers can find descriptive information of the sub-cohorts included in DLCC, such as the introduction of datasets, methods used in collecting data, data quality control strategies, methods for statistical analyses, relevant publications, etc. In brief, a multistage stratified cluster sampling procedure was used to enroll the participants. In stage one, we selected districts in Guangdong Province, and then districts or counties in the cities of Guangdong were selected. In the third stage, communities were selected from urban streets or districts, and villages were selected from counties. Finally, residents living in the selected communities and villages were invited to participate in the baseline survey.

Eligible participants were aged 18 years and older, had local household registration, and had resided in the area for at least six months prior to the survey. Exclusion criteria included pregnancy, severe mental or physical conditions, and active-duty military personnel. A total of 8,177 eligible individuals were enrolled in the sleep study. Of the 8,177 eligible participants enrolled at baseline, 1,645 were excluded due to incomplete sleep monitoring data (including 664 who refused the test, 186 with lost or damaged monitors, and 795 with invalid data). Among the remaining 6,532 participants with complete sleep monitoring data, an additional 1,013 were excluded due to missing fracture history (*n* = 98) or missing covariates required for the multivariable regression models (*n* = 915), resulting in a final analytical sample of 5,519 participants (e-Figure 1, supplementary material 2).

This study was conducted in accordance with the ethical principles of the Declaration of Helsinki. The study protocol was reviewed and approved by the Ethics Committee of the Guangdong Provincial People’s Hospital. All the participants provided written informed consent.

### Data collection

The baseline data were collected by trained personnel according to standard operating procedures. Face-to-face questionnaires (questionnaires were developed for this study, see supplementary material 1) were used to collect information on sociodemographic characteristics, personal medical history, and physical activity. The completeness and accuracy of each questionnaire were checked by an epidemiologist through a face-to-face review of the subjects. The physical examination included weight, height, grip, and blood pressure. Body weight, height and waist circumference were measured according to standard protocols. BMI was calculated as weight/height squared (kg/m^2^) and divided into three groups: normal, < 25, overweight 25–30, and obese, ≥ 30. Blood pressure was measured three consecutive times in a seated position after at least 5 min of rest using a digital sphygmomanometer. Grip strength was measured using a hand dynamometer (Jamar Plus+, Shanghai, China) for the dominant hand. Each measurement was repeated twice, and the higher value was recorded. Fasting venous blood samples (≥ 8 h) were obtained from all participants. Hemoglobin, serum creatinine, blood glucose, and serum lipid levels were measured.

### Sleep study and assessment of OSA

Participants were required to undergo an in-home sleep monitoring test using a Type-IV Wearable Intelligent Sleep Monitor (WISM, Chengdu, China). The WISM, with dimensions of only 29 × 23 × 10 mm and a weight of just 5.35 g, is attached to the thenar eminence of the palm using medical double-sided adhesive (e-Figure 2, supplementary material 2). It automatically records and analyzes the duration of effective monitoring and blood oxygen saturation.

The device operates based on the photoplethysmography principle, which measures blood volume in the skin’s microvascular layer by exploiting the absorption and reflection of specific light wavelengths by human tissue. Deoxygenated hemoglobin absorbs more red wavelengths, while oxygenated hemoglobin absorbs more infrared wavelengths [15]. Utilizing a photoreflective sensor, pulse oximetry is measured based on the absorption characteristics of hemoglobin for infrared and red light, and the raw data is stored in a specialized database.

The primary oximetric indices monitored included the oxygen desaturation index (ODI), minimum oxygen saturation, the percentage of sleep time spent with SpO2 < 90% (CT90%), and cumulative time at SpO2 < 90% (TS90%). The ODI is defined as the number of events per hour in which oxygen saturation decreases by ≥ 3% from baseline.

The validity of pulse-oximetry using synchronous overnight recordings of both Type-IV WISM and standard polysomnography (PSG) was confirmed among 196 patients in a sleep disordered breathing center [16]. The sensitivity and specificity were 93% and 77%, respectively, for detecting an apnea-hypopnea index (AHI) of ≥ 5 by PSG using a cut-off threshold of 3% ODI = 5. Similarly, the sensitivity and specificity for detecting AHI of ≥ 15 by PSG using a cut-off threshold of 3% ODI = 15 were 92% and 89%, respectively. Furthermore, the ODI has been validated as a potential screening tool for OSA in various populations [17–20].

This study employed clinically established AHI thresholds to define OSA severity: ODI < 5 indicates no OSA, ≥ 5 and < 15 as mild, ≥ 15 as moderate to severe [21].

### Assessment criteria of fractures and covariates

A history of past fractures, including non-traumatic and traumatic fractures, was recorded at the baseline visit if the participant self-reported a previous fracture diagnosed by a certified physician. Thus, the fracture data represents retrospectively ascertained events that predated the in-home sleep test for OSA. Hypertension and diabetes were defined based on a self-reported history or current use of therapeutic medications. Cardiovascular disease history was defined as self-reported occurrences of ischemic and hemorrhagic cerebrovascular accidents, transient ischemic attacks, acute myocardial infarction, post-coronary stent implantation, post-coronary artery bypass grafting, angina pectoris, thromboembolic diseases, or cardiac arrest. Smoking was defined as continuous or cumulative smoking for more than six months. Alcohol consumption was defined as the consumption of ≥ 2 drinks per month. Poor sleep quality was defined as a Pittsburgh Sleep Quality Index score >5 [22].

### Statistical analysis

Baseline characteristics are presented as the mean ± SD for continuous variables and as numbers (%) for categorical variables. Student’s t-test or the Mann-Whitney U test was used to assess significant differences for continuous variables and the χ2 test for categorical variables.

All participants were divided into groups according to tertiles of age as follows: T1 group, 18–47 years; T2 group, 48–58 years; T3 group, 59–91 years. Multivariable logistic regression models (odds ratio (OR) and 95% confidence interval [95% CIs]) were used to assess the association between OSA and the prevalence of self-reported history of fractures, with and without adjustment for potential covariates. Trend tests were performed by modeling the differential ODI categories as continuous variables. We used models with restricted cubic splines to explore associations between OSA and self-reported history of fractures after adjusting for potential confounders. Moreover, we stratified the population by age tertiles, sex, race, current drinking (no vs. yes), current smoking (no vs. yes), BMI (normal vs. overweight or obesity), and poor sleep (no vs. yes) to assess the sensitivity and interactions within different subgroups.

All data analyses were performed using EmpowerStats (http://www.empowerstats.net/cn/index.php) and R software, version 4.2.3 (http://www.r-project.org). A two-tailed *P* value of less than 0.05 was considered statistically significant.

## Results

### Characteristics of the study participants

Table 1 shows the characteristics of the study participants. The mean age of participants was 52.7 years. The prevalence of current smoking and drinking among participants was 13.3% and 16.7%, respectively. Additionally, the prevalence of self-reported history of fractures, OSA, hypertension, diabetes, tumors, overweight, and obesity was 13%, 44.4%, 18.6%, 8.1%, 2.5%, 29.9%, and 3.9%, respectively. Most participants in this study had poor sleep quality (53.2%). Participants in the higher age tertile (59–91 years) were more likely to be male and had a higher proportion of current smoking, regular exercise, self-reported history of fractures, history of tumor, history of CVD, overweight, OSA, and poor sleep quality, along with higher systolic blood pressure, diastolic blood pressure, hemoglobin, fasting glucose, total cholesterol, triglycerides, ODI, and CT 90%, and lower grip strength, as compared to participants in tertile 1 (18–47 years) or 2 (48–58 years).

**Table 1:** Baseline characteristics of the study participants according to age tertiles

Variable	Total	Age tertiles			*P*-value
		Tertile 1 (18–47 years)	Tertile 2 (48–58 years)	Tertile 3 (59–91 years)	
Participants, no.(%)	5519	1770	1909	1840	
Age, year	52.7 ± 12.6	38.1 ± 6.8	53.3 ± 3.1	66.3 ± 5.7	< 0.001
Men, no.(%)	1690 (30.6)	455 (25.7)	551 (28.9)	684 (37.2)	< 0.001
Minority, no.(%)	753 (13.6)	293 (16.6)	249 (13.0)	211 (11.5)	< 0.001
Urban residents, no.(%)	4060 (73.6)	1246 (70.4)	1421 (74.4)	1393 (75.7)	< 0.001
Marital status, no.(%)					< 0.001
Unmarried	259 (4.7)	240 (13.6)	11 (0.6)	8 (0.4)	
Married	4815 (87.2)	1482 (83.7)	1794 (94.0)	1539 (83.6)	
Divorced or widowed	445 (8.1)	48 (2.7)	104 (5.4)	293 (15.9)	
Education, no.(%)					< 0.001
Middle school or below	2737 (49.6)	510 (28.8)	1096 (57.4)	1131 (61.5)	
High school	1321 (23.9)	342 (19.3)	419 (21.9)	560 (30.4)	
College or above	1461 (26.5)	918 (51.9)	394 (20.6)	149 (8.1)	
Smoking status, no.(%)					< 0.001
Never	4550 (82.4)	1555 (87.9)	1584 (83.0)	1411 (76.7)	
Ever	235 (4.3)	26 (1.5)	58 (3.0)	151 (8.2)	
Current	734 (13.3)	189 (10.7)	267 (14.0)	278 (15.1)	
Drinking status, no.(%)					< 0.001
Never	4457 (80.8)	1454 (82.1)	1532 (80.3)	1471 (79.9)	
Ever	143 (2.6)	22 (1.2)	39 (2.0)	82 (4.5)	
Current	919 (16.7)	294 (16.6)	338 (17.7)	287 (15.6)	
Regular exercise, no.(%)	3161 (57.3)	733 (41.4)	1164 (61.0)	1264 (68.7)	< 0.001
Reported previous fractures, no.(%)	720 (13.0)	156 (8.8)	241 (12.6)	323 (17.6)	< 0.001
Type of reported previous fractures, no.(%)					0.237
Traumatic fractures	558 (82.8)	123 (83.1)	190 (86.0)	245 (80.3)	
Non-traumatic fractures	116 (17.2)	25 (16.9)	31 (14.0)	60 (19.7)	
Hypertension, no.(%)	1025 (18.6)	90 (5.1)	356 (18.6)	579 (31.5)	< 0.001
Diabetes, no.(%)	449 (8.1)	25 (1.4)	143 (7.5)	281 (15.3)	< 0.001
History of tumor, no.(%)	138 (2.5)	34 (1.9)	47 (2.5)	57 (3.1)	0.076
History of CVD, no.(%)	137 (2.5)	10 (0.6)	30 (1.6)	97 (5.3)	< 0.001
Body mass index, no.(%)					< 0.001
Normal weight	3658 (66.3)	1252 (70.7)	1226 (64.2)	1180 (64.1)	
Overweight	1648 (29.9)	445 (25.1)	607 (31.8)	596 (32.4)	
Obesity	213 (3.9)	73 (4.1)	76 (4.0)	64 (3.5)	
Systolic blood pressure, mmHg	125.5 ± 19.0	115.0 ± 15.0	127.0 ± 17.7	134.3 ± 18.9	< 0.001
Diastolic blood pressure, mmHg	75.5 ± 11.4	72.3 ± 11.0	77.6 ± 11.3	76.6 ± 11.1	< 0.001
Grip, kg	30.0 ± 9.5	32.2 ± 9.8	30.2 ± 9.2	27.6 ± 8.8	< 0.001
Hemoglobin, g/L	141.4 ± 15.3	138.9 ± 17.2	142.5 ± 14.2	142.8 ± 14.0	< 0.001
Serum creatinine, µmol/L	70.8 ± 23.6	66.4 ± 15.1	70.0 ± 27.7	76.0 ± 24.8	< 0.001
Fasting glucose, mmol/L	5.9 ± 1.6	5.3 ± 1.0	5.9 ± 1.7	6.4 ± 1.8	< 0.001
Total cholesterol, mmol/L	5.5 ± 1.1	5.2 ± 1.0	5.7 ± 1.1	5.7 ± 1.2	< 0.001
Triglycerides, mmol/L	1.6 ± 1.3	1.4 ± 1.3	1.7 ± 1.4	1.6 ± 1.0	< 0.001
LDL cholesterol, mmol/L	3.2 ± 0.9	2.9 ± 0.8	3.3 ± 0.9	3.3 ± 1.0	< 0.001
ODI, events/h	7.1 ± 7.8	6.3 ± 7.6	6.6 ± 7.4	8.4 ± 8.4	< 0.001
OSA groups, events/h					< 0.001
Normal	3068 (55.6)	1122 (63.4)	1106 (57.9)	840 (45.7)	
Mild	1714 (31.1)	440 (24.9)	595 (31.2)	679 (36.9)	
Moderate to severe	737 (13.4)	208 (11.8)	208 (10.9)	321 (17.4)	
Average oxygen saturation, (%)	96.5 ± 1.7	97.0 ± 1.7	96.5 ± 1.5	96.0 ± 1.7	< 0.001
Minimum oxygen saturation, (%)	86.1 ± 5.8	86.6 ± 6.1	86.4 ± 5.3	85.4 ± 5.9	< 0.001
CT 90, (%)	2.1 ± 7.6	1.9 ± 7.7	1.8 ± 6.7	2.6 ± 8.3	0.002
Poor sleep, no.(%)	2533 (53.2)	708 (46.6)	876 (52.8)	949 (59.9)	< 0.001

*Abbreviation*: *CT90%* percentage of sleep time spent with SpO2 < 90%, *CVD* cardiovascular diseases, *LDL* low-density lipoprotein, *OSA* obstructive sleep apnea, *ODI* oxygen desaturation index

Data are weighted and expressed as mean ± SD and number (%)

### Association between self-reported history of fractures and OSA according to age tertiles

**Fig. 1:**
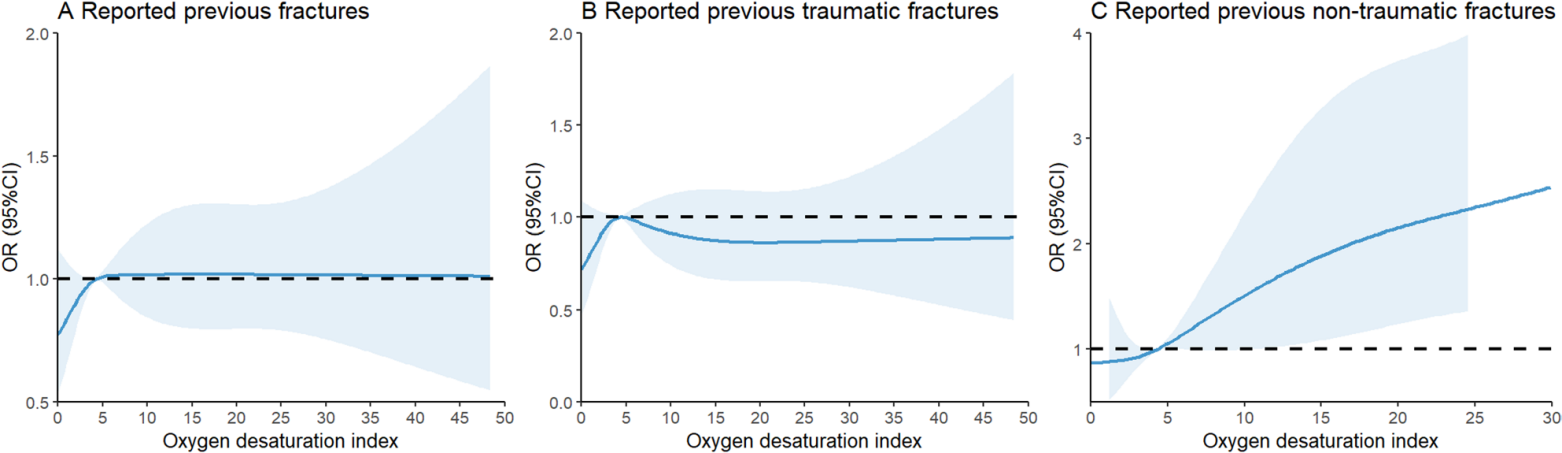
The relationship between the self-reported previous fractures (**A**), the self-reported previous traumatic fractures (**B**) and self-reported previous non-traumatic fractures (**C**) and ODI Adjusted for sex, race, township, marriage status, education, drinking, smoking, hypertension, diabetes, history of tumor, history of cardiovascular diseases, body mass index, waist circumference, systolic blood pressure, diastolic blood pressure, regular exercise, triglycerides, total cholesterol, and grip. Abbreviations: ODI: oxygen desaturation index

**Fig. 2:**
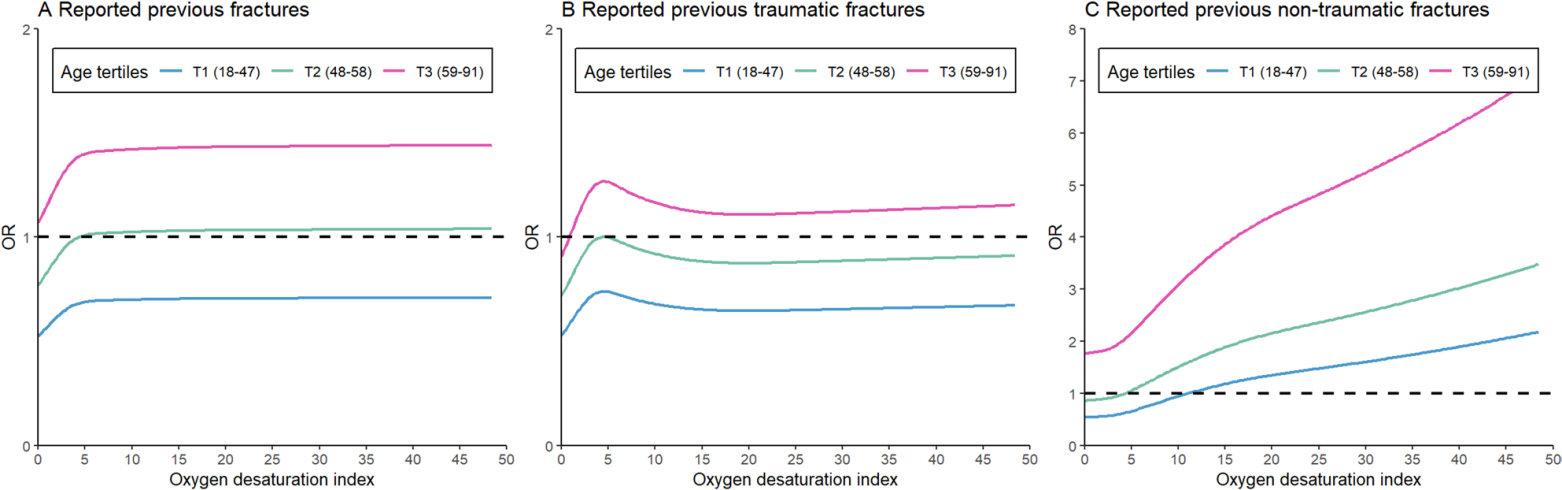
The relationship between the self-reported previous fractures (**A**), the self-reported previous traumatic fractures (**B**) and self-reported previous non-traumatic fractures (**C**) and ODI by categories of age tertiles Adjusted for sex, race, township, marriage status, education, drinking, smoking, hypertension, diabetes, history of tumor, history of cardiovascular diseases, body mass index, waist circumference, systolic blood pressure, diastolic blood pressure, regular exercise, triglycerides, total cholesterol, and grip. Abbreviations: ODI: oxygen desaturation index

The association between the ODI and self-reported history of fractures according to tertiles of age is shown in Figs. 1 and 2. Table 2 shows the results of the multivariate regression models. Overall, a significant trend was observed across OSA groups, indicating that higher OSA severity was associated with increased odds of self-reported previous non-traumatic fractures (P for trend < 0.001). Among participants in age tertile 3 (59–91 years), mild (OR = 1.36, 95% CI: 1.02–1.79) and moderate to severe OSA (OR = 1.45, 95% CI: 1.01–2.08) were associated with 36% and 45% higher odds of self-reported previous fractures, respectively, compared with those without OSA. A significant stepwise increase in self-reported history of fractures odds was observed across OSA groups in the group of age tertile 3 (P for trend = 0.019). Similar results were observed for non-traumatic fractures. However, OSA was not associated with self-reported previous traumatic fractures (OR = 1.14, 95%CI: 0.75–1.73). Among participants in age tertile 2 (48–58 years), only moderate to severe OSA was associated with increased odds of self-reported previous non-traumatic fractures (OR = 2.92, 95% CI: 1.06–8.02) compared with those without OSA. Furthermore, OSA was not associated with any self-reported history of fractures (P for trend > 0.1) in participants in age tertile 1 (18–47 years).

**Table 2:** The associations between OSA and self-reported history of fractures stratified by age tertiles*

Exposure	Total	*P*-value	Age tertile 1 (18–47 years)	*P*-value	Age tertile 2 (48–58 years)	*P*-value	Age tertile 3 (59–91 years)	*P*-value
Self-reported previous fractures								
OSA groups,								
Normal	Ref.		Ref.		Ref.		Ref.	
Mild	1.07 (0.89, 1.28)	0.481	0.72 (0.44, 1.20)	0.21	0.99 (0.72, 1.35)	0.945	1.36 (1.02, 1.79)	0.033
Moderate to severe	1.08 (0.84, 1.39)	0.529	0.92 (0.49, 1.75)	0.804	0.95 (0.59, 1.54)	0.842	1.45 (1.01, 2.08)	0.044
P for trend	0.442		0.502		0.855		0.019	
Self-reported previous traumatic fractures								
OSA groups,								
Normal	Ref.		Ref.		Ref.		Ref.	
Mild	0.98 (0.80, 1.21)	0.882	0.68 (0.39, 1.19)	0.182	0.93 (0.65, 1.31)	0.665	1.25 (0.92, 1.71)	0.153
Moderate to severe	0.90 (0.67, 1.20)	0.459	0.90 (0.45, 1.80)	0.758	0.79 (0.45, 1.37)	0.395	1.14 (0.75, 1.73)	0.531
P for trend	0.511		0.452		0.396		0.328	
Self-reported previous non-traumatic fractures								
OSA groups,								
Normal	Ref.		Ref.		Ref.		Ref.	
Mild	1.39 (0.88, 2.18)	0.156	0.74 (0.23, 2.30)	0.598	1.05 (0.43, 2.56)	0.915	1.99 (1.04, 3.81)	0.037
Moderate to severe	2.65 (1.60, 4.41)	< 0.001	1.58 (0.46, 5.36)	0.465	2.92 (1.06, 8.02)	0.037	3.68 (1.79, 7.57)	< 0.001
P for trend	< 0.001		0.695		0.081		< 0.001	

*Adjusted variables included sex, race, township, marriage status, education, drinking, smoking, hypertension, diabetes, history of tumor, history of cardiovascular diseases, body mass index, waist circumference, systolic blood pressure, diastolic blood pressure, regular exercise, triglycerides, total cholesterol, and grip

### Association between self-reported history of fractures and comorbid OSA and poor sleep quality according to age tertiles

After adjusting for potential confounders, participants with both OSA and poor sleep quality in age tertile 3 (59–91 years) were significantly associated with 53% higher odds of self-reported previous fractures than those with OSA alone (OR = 1.53, 95%CI:1.01–2.31) (Table 3). No significant associations were observed in the first (18–47 years) and second (48–58 years) age tertiles. Similar results were observed in self-reported previous traumatic fractures. However, the combination of OSA and poor sleep quality did not significantly increase the odds of self-reported previous non-traumatic fractures compared with OSA alone.

**Table 3:** Association of OSA and poor sleep quality with self-reported history of fractures stratified by age tertiles*

Exposure	Total	*P*-value	Age tertile 1 (18–47 years)	*P*-value	Age tertile 2 (48–58 years)	*P*-value	Age tertile 3 (59–91 years)	*P*-value
Self-reported previous fractures								
OSA alone	Ref.		Ref.		Ref.		Ref.	
OSA with poor sleep	1.20 (0.92, 1.56)	0.178	1.02 (0.56, 1.88)	0.939	0.80 (0.51, 1.26)	0.339	1.53 (1.01, 2.31)	0.045
Self-reported previous traumatic fractures								
OSA alone	Ref.		Ref.		Ref.		Ref.	
OSA with poor sleep	1.32 (0.98, 1.79)	0.069	0.98 (0.49, 1.97)	0.96	0.91 (0.55, 1.53)	0.731	1.64 (1.02, 2.65)	0.041
Self-reported previous non-traumatic fractures								
OSA alone	Ref.		Ref.		Ref.		Ref.	
OSA with poor sleep	0.98 (0.55, 1.77)	0.955	1.29 (0.29, 5.78)	0.737	0.46 (0.16, 1.34)	0.153	1.59 (0.65, 3.86)	0.309

*Adjusted variables included sex, race, township, marriage status, education, drinking, smoking, hypertension, diabetes, history of tumor, history of cardiovascular diseases, body mass index, waist circumference, systolic blood pressure, diastolic blood pressure, regular exercise, triglycerides, total cholesterol, and grip

### Stratified analyses

**Fig. 3:**
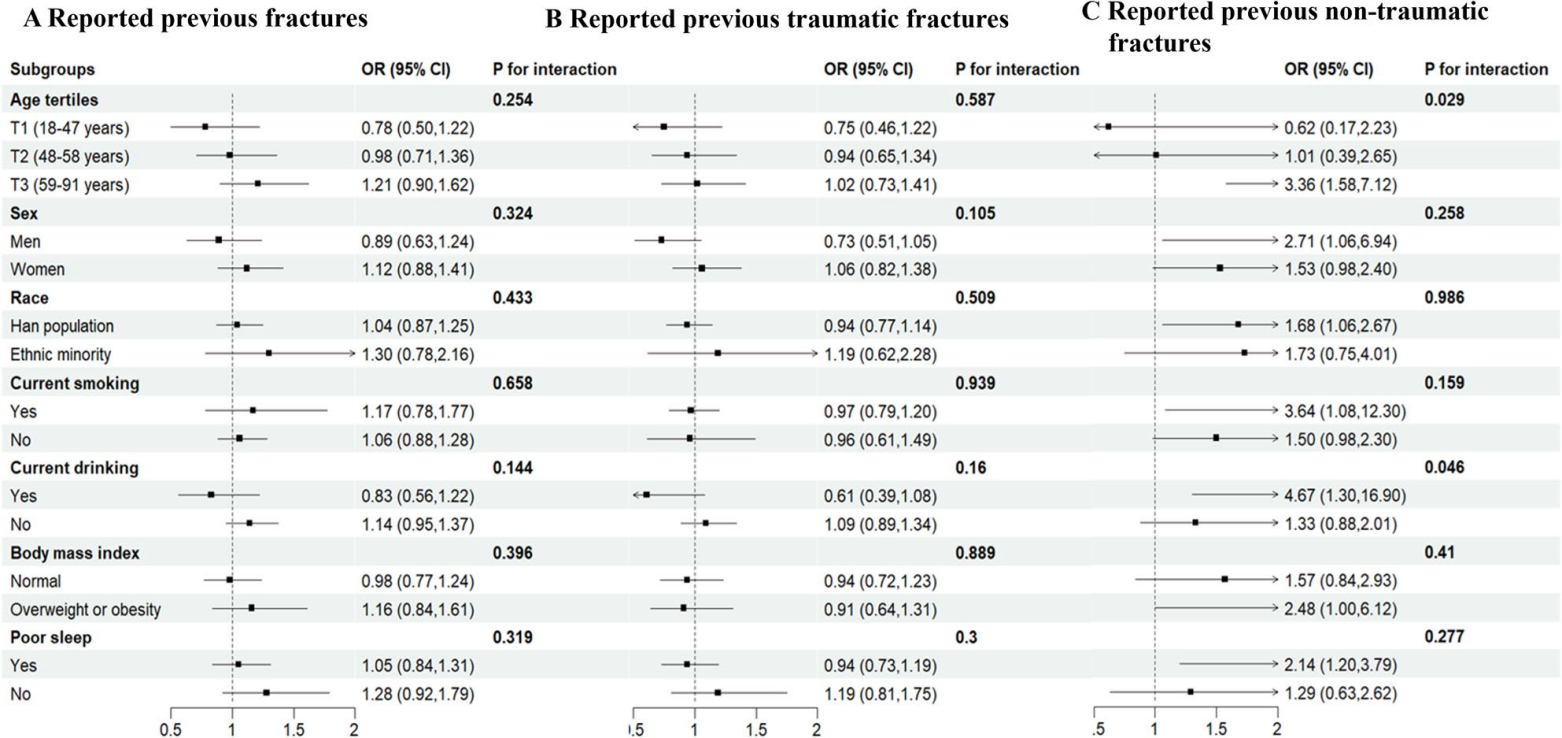
Stratified analysis of the OSA (yes vs. no) and the self-reported previous fractures (**A**) the self-reported previous traumatic fractures (**B**) and self-reported previous non-traumatic fractures (**C**) Adjusted for sex, race, township, marriage status, education, drinking, smoking, hypertension, diabetes, history of tumor, history of cardiovascular diseases, body mass index, waist circumference, systolic blood pressure, diastolic blood pressure, regular exercise, triglycerides, total cholesterol, and grip. Abbreviations: OSA: obstructive sleep apnea

Stratified analyses were performed to assess the association between OSA and self-reported history of fractures across various subgroups (Fig. 3). This study identified significant interactions between OSA and age tertiles (P for interaction = 0.029), as well as between OSA and alcohol consumption concerning self-reported previous non-traumatic fractures (P for interaction = 0.046). None of these variables, including sex, race, smoking, body mass index, and poor sleep quality, significantly modified the association between OSA and self-reported history of fractures (P for interaction > 0.05 for all comparisons).

## Discussion

To our knowledge, this is the first study to investigate the association between OSA and self-reported history of fractures in the general population. Our results indicate that OSA was only associated with higher odds of non-traumatic fractures in middle-aged and older participants. Additionally, we found that OSA interacted with both age and drinking to influence the odds of self-reported previous fractures and non-traumatic fractures, respectively. Our findings suggest that patients with OSA, particularly older individuals, those who consume alcohol, and those with poor sleep quality, should receive particular attention regarding fracture-related risks, with appropriate preventive measures implemented.

Current research on the impact of OSA on bone health remains limited and has yielded inconsistent conclusions. While the majority of existing studies suggest a significant association between OSA and bone mineral density (BMD) [23], some report no significant correlation [24, 25], and others even indicate an association between OSA and increased BMD [26, 27]. A recent meta-analysis involving 158,427 participants demonstrated that OSA is associated with lower BMD, elevated levels of certain bone resorption markers, and an increased risk of osteoporosis [28]. It is well-established that BMD is closely related to age; human BMD typically peaks around the age of 40, followed by a gradual decline, which becomes more pronounced in postmenopausal women and individuals over 60 [29]. The discrepancies in current research findings may be attributed to several factors, including differences in bone metabolism characteristics across age groups, varying tolerance to chronic intermittent hypoxia, heterogeneity in OSA duration, inadequate control for confounding variables, and variations in study design. Our findings indicate that a positive association between the ODI and self-reported history of fractures was observed in the middle-aged and elderly community population, with no such correlation identified in younger adults.

Chronic nocturnal intermittent hypoxia is a hallmark of OSA; however, the skeletal response to OSA-related hypoxia in humans remains poorly understood. A major challenge is that patients with OSA experience recurrent nocturnal hypoxemia of varying severity, marked by repeated cycles of desaturation and reoxygenation, which are difficult to replicate accurately in vitro. Consequently, most studies on the relationship between OSA and bone metabolism have relied on human cell cultures or animal models, such as mice. Therefore, the possible explanations for the findings in our study remain to be further elucidated; we speculate that there are several potential mechanisms that may explain the results of this study.

First, the human skeleton is renewed and regenerated throughout life. Under normal conditions, bone mass is maintained by a balanced activity of osteoclasts and osteoblasts. These actions are coordinated by osteocytes – the “master regulators” of bone remodeling that coordinate the actions of osteoblasts and osteoclasts through, for example, secretion of endocrine and signaling factors and by acting as mechanosensory cells [30, 31]. However, with advancing age, there is a reduction in osteoblasts and a decline in their function, coupled with an increase in osteoclast activity and a shift in mesenchymal stem cell differentiation toward adipocytes [32]. Furthermore, the aging process is accompanied by a chronic low-grade inflammatory state [33] and increased oxidative stress, which have been demonstrated in numerous animal studies [34]. These age-related alterations in the bone marrow microenvironment disrupt normal bone remodeling, resulting in decreased bone formation and enhanced bone resorption.

Moreover, in the elderly, the prevalence of OSA is about three to four times higher than in younger adults [35], and the disease course is often longer. Older adults with OSA have a significantly greater nocturnal hypoxic burden than younger individuals [36]. In this study, both average and minimum oxygen saturation levels were significantly lower in older adults than in younger participants. Prolonged and untreated chronic nocturnal intermittent hypoxia may exacerbate inflammation and oxidative stress in the bone marrow microenvironment of the elderly, leading to microenvironmental acidosis [7]. Epidemiological evidence indicates that inflammatory changes in the bone microenvironment are associated with an increased risk of fractures [9, 10]. In addition, the aging skeletal system exhibits a reduction in the vascular niches that support hematopoietic stem cells [37]. Hypoxia may further worsen this condition by inducing endothelial dysfunction and impairing angiogenesis, ultimately reducing bone perfusion and accelerating bone loss [38]. Therefore, under comparable hypoxic conditions, hypoxia-induced impairment of bone remodeling is likely more severe in older OSA patients than in younger individuals.

Second, adequate sleep aligned with the circadian night is essential for multiple biological processes and systems. Circadian disruptions and alterations in the timing and duration of sleep are associated with numerous metabolic [39], cardiovascular [40], endocrine [41], and neurological disorders [42]. Furthermore, the daily rhythm in bone turnover markers [43, 44], the existence of clock genes in bone cells, the identification of altered skeletal phenotypes in clock gene knockout models [45–48], and the discovery that repeated sleep restriction arrests bone remodeling in laboratory rats [49], all indicate that disruptions in the physiology of sleep and circadian rhythmicity may also affect bone health.

Recurrent episodes of apnea or hypopnea in patients with OSA cause sleep fragmentation, leading to accumulated sleep loss or “sleep debt”. Compared with younger and middle-aged adults, older adults generally have shorter total sleep duration and reduced quantity and quality of deep sleep, particularly slow-wave sleep (SWS) [50]. SWS is associated with increased parasympathetic activity, decreased sympathetic activity, and enhanced release of certain hormones, including growth hormone [7]. OSA-related sleep fragmentation further exacerbates the already shortened sleep duration in the elderly and disrupts their limited physiological sleep recovery processes during sleep. Consequently, the circadian rhythm of bone turnover may be more vulnerable to OSA-related disruption in older adults than in younger populations.

Furthermore, evidence suggests that melatonin plays a key role in promoting bone formation and inhibiting bone resorption [51]. Melatonin secretion decreases with age, leading to lower levels in older adults compared to younger individuals [52]. However, it remains unclear whether melatonin levels or rhythmicity differ between OSA patients and the general population. Individuals with OSA are more likely to be exposed to light at night, which may theoretically disrupt melatonin secretion—known to follow a circadian pattern and be inhibited by retinal light exposure—thereby interfering with bone remodeling. Future studies are warranted to clarify the relationship between melatonin levels and bone health in OSA patients.

Overall, current evidence indicates that physiologic and metabolic disturbances in OSA are linked to increased bone resorption and, consequently, lower BMD. This association appears to be more pronounced in older adults than in younger individuals.

Recurrent nocturnal hypoxia in OSA may cause repeated nighttime awakenings, thereby disrupting normal sleep physiology. Epidemiological studies suggest that an OSA subtype characterized by sleep disturbances accounts for about 32.7% of patients with OSA [53]. Our findings indicate that individuals with both OSA and poor sleep quality have significantly higher odds of reporting previous traumatic fractures than those with OSA alone. A prospective cohort study of 2,911 elderly men showed that, after an average follow-up of 6.8 years, those with ≥ 10% of sleep time at < 90% oxygen saturation had a 25% higher risk of at least one fall and a 43% higher risk of two or more falls compared with those with < 1% of sleep time below this threshold [13].

Notably, the prevalence of poor sleep quality in this study was 53.2%, exceeding previously self-reported rates in Chinese populations, which ranged from 16.4% to 39.4% [54]. This discrepancy may be largely attributable to the demographic characteristics of our study population, which consisted predominantly of middle-aged and older adults, with women comprising 69.4% of the sample. Epidemiological studies have shown that the prevalence of insomnia increases with age [55]. Moreover, women are approximately 1.5 times more likely than men to experience insomnia, likely due to hormonal changes during the peri- and postmenopausal periods [56].

These findings underscore the importance of prioritizing sleep quality in older adults with OSA, particularly women. Early interventions and preventive strategies may help reduce the risk of traumatic fractures in this population.

Our study identified an interaction between alcohol consumption and OSA regarding self-reported previous non-traumatic fractures, suggesting that OSA patients who consume alcohol have significantly higher odds of self-reported previous non-traumatic fractures compared to non-drinkers. Previous studies have shown that alcohol consumption can prolong apnea duration and worsen OSA-related hypoxemia [57], both of which are associated with a higher risk of fractures [13]. In addition, excessive alcohol consumption independently increases fracture risk, whereas the effects of moderate or low-dose intake remain unclear [58]. Further research is warranted to determine whether alcohol dose modifies the relationship between OSA and non-traumatic fracture risk.

### Strengths and limitations

The strengths of this study include its ability to examine the relationship between OSA and self-reported history of fractures in a large sample size.

This study has several limitations. First, fractures were self-reported to have occurred before OSA assessment, and the cross-sectional design limits the ability to infer causality. Reverse causation also cannot be excluded (e.g., reduced physical activity after a fracture leading to weight gain and subsequent OSA).

Nevertheless, several findings support the biological plausibility of OSA as a contributing risk factor. First, OSA is a chronic condition that develops gradually and often remains undiagnosed for years. Thus, many participants diagnosed with OSA at baseline were likely already affected by the disorder—and its adverse effects on bone metabolism—when their fractures occurred. Second, and more importantly, the significant association observed in older adults—who are more prone to have poorer bone quality—aligns with the proposed pathophysiological mechanism above. The lack of an association among younger, more resilient participants argues against reverse causation as the sole explanation and supports the hypothesis that OSA interacts with age-related vulnerabilities to elevate fracture risk. Further prospective cohort studies are warranted to confirm these findings.

Another limitation is the lack of baseline data on fracture sites, which prevented analysis of potential site-specific associations with OSA. Additional studies are needed to address this issue. Moreover, the small number of participants aged ≥ 80 years (*n* = 52) limited our ability to perform a more detailed age-stratified analysis. Future studies with larger samples of very old participants are required to validate and refine our findings in this vulnerable population.

Furthermore, data on medications that may increase fracture risk (e.g., corticosteroids) were unavailable in this cohort. Although we adjusted for a wide range of comorbidities and clinical risk factors, residual confounding by unmeasured medication use cannot be entirely ruled out. Finally, PSG remains the gold standard for diagnosing OSA. Although OSA was assessed using a wearable sleep monitor in this study, previous research has demonstrated good agreement between this method and PSG [16].

## Conclusion

In summary, OSA was significantly associated only with self-reported previous non-traumatic fractures in the general population. The association was stronger in middle-aged and older individuals but not in younger population. Interactions between OSA and age, as well as between OSA and alcohol consumption, regarding self-reported previous non-traumatic fractures were found in this study. Additionally, OSA patients with poor sleep quality had higher odds of self-reported previous traumatic fractures. Further prospective cohort study is warranted to verify our findings.

## Supplementary Information

Supplementary Material 1.

Supplementary Material 2.

## Data Availability

Data will be made available only to potential collaborators with ethical approval after they submit a research proposal application by contacting the corresponding author.

## Abbreviations

BMD: Bone mineral density
CT90%: Percentage of sleep time spent with SpO2 < 90%
LDL-C: Low-density lipoprotein cholesterol
ODI: Oxygen desaturation index
OSA: Obstructive sleep apnea
SWS: Slow-wave sleep
SD: Standard deviation
