# Correction: Age-dependent association between obstructive sleep apnea and self-reported history of fractures: a community-based study

**DOI:** 10.1186/s12889-026-27021-z

**Published:** 2026-04-13

**Authors:** Junzhi Chen, Guangliang Shan, Yaoda Hu, Huijing He, Tong Feng, Ruohan Zhou, Ping Yuan, Miaochan Lao, Qiong Ou

**Affiliations:** 1 Department of Respiratory and Critical Care Medicine, Sleep Center, Guangdong Provincial People’s Hospital, Guangdong Academy of Medical Sciences, Southern Medical University, 106 Zhongshan Second Road, Yuexiu District, Guangzhou, 510080 China; 2 Department of Epidemiology and Statistics, Institute of Basic Medical Sciences, School of Basic Medicine, Chinese Academy of Medical Sciences, Peking Union Medical College, Beijing, China

**Correction: BMC Public Health 25**,** 4291 (2025)**

**https://doi.org/10.1186/s12889-025-25593-w**

Following publication of the original article [1], the authors identified two errors that require correction:

1. In the author affiliations, the institutional name was incorrectly listed as “Guangdong Provincial Peoples Hospital”. The correct name should be “Guangdong Provincial People’s Hospital”.
2. In Table 1. The units for the following three variables are listed as mg/dL, while the correct unit should be mmol/L:
  1. Total cholesterol
  2. Triglycerides
  3. LDL cholesterol

The correct Table 1 is presented below.

**Table 1:** Baseline characteristics of the study participants according to age tertiles

Variable	Total	Age tertiles			P-value
		Tertile 1 (18-47 years)	Tertile 2 (48-58 years)	Tertile 3 (59-91 years)	
Participants, no.(%)	5519	1770	1909	1840	
Age, year	52.7 ± 12.6	38.1 ± 6.8	53.3 ± 3.1	66.3 ± 5.7	<0.001
Men, no.(%)	1690 (30.6)	455 (25.7)	551 (28.9)	684 (37.2)	<0.001
Minority, no.(%)	753 (13.6)	293 (16.6)	249 (13.0)	211 (11.5)	<0.001
Urban residents, no.(%)	4060 (73.6)	1246 (70.4)	1421 (74.4)	1393 (75.7)	<0.001
Marital status, no.(%)					<0.001
Unmarried	259 (4.7)	240 (13.6)	11 (0.6)	8 (0.4)	
Married	4815 (87.2)	1482 (83.7)	1794 (94.0)	1539 (83.6)	
Divorced or widowed	445 (8.1)	48 (2.7)	104 (5.4)	293 (15.9)	
Education, no.(%)					<0.001
Middle school or below	2737 (49.6)	510 (28.8)	1096 (57.4)	1131 (61.5)	
High school	1321 (23.9)	342 (19.3)	419 (21.9)	560 (30.4)	
College or above	1461 (26.5)	918 (51.9)	394 (20.6)	149 (8.1)	
Smoking status, no.(%)					<0.001
Never	4550 (82.4)	1555 (87.9)	1584 (83.0)	1411 (76.7)	
Ever	235 (4.3)	26 (1.5)	58 (3.0)	151 (8.2)	
Current	734 (13.3)	189 (10.7)	267 (14.0)	278 (15.1)	
Drinking status, no.(%)					<0.001
Never	4457 (80.8)	1454 (82.1)	1532 (80.3)	1471 (79.9)	
Ever	143 (2.6)	22 (1.2)	39 (2.0)	82 (4.5)	
Current	919 (16.7)	294 (16.6)	338 (17.7)	287 (15.6)	
Regular exercise, no.(%)	3161 (57.3)	733 (41.4)	1164 (61.0)	1264 (68.7)	<0.001
Reported previous fractures, no.(%)	720 (13.0)	156 (8.8)	241 (12.6)	323 (17.6)	<0.001
Type of reported previous fractures, no.(%)					0.237
Traumatic fractures	558 (82.8)	123 (83.1)	190 (86.0)	245 (80.3)	
Non-traumatic fractures	116 (17.2)	25 (16.9)	31 (14.0)	60 (19.7)	
Hypertension, no.(%)	1025 (18.6)	90 (5.1)	356 (18.6)	579 (31.5)	<0.001
Diabetes, no.(%)	449 (8.1)	25 (1.4)	143 (7.5)	281 (15.3)	<0.001
History of tumor, no.(%)	138 (2.5)	34 (1.9)	47 (2.5)	57 (3.1)	0.076
History of CVD, no.(%)	137 (2.5)	10 (0.6)	30 (1.6)	97 (5.3)	<0.001
Body mass index, no.(%)					<0.001
Normal weight	3658 (66.3)	1252 (70.7)	1226 (64.2)	1180 (64.1)	
Overweight	1648 (29.9)	445 (25.1)	607 (31.8)	596 (32.4)	
Obesity	213 (3.9)	73 (4.1)	76 (4.0)	64 (3.5)	
Systolic blood pressure, mmHg	125.5 ± 19.0	115.0 ± 15.0	127.0 ± 17.7	134.3 ± 18.9	<0.001
Diastolic blood pressure, mmHg	75.5 ± 11.4	72.3 ± 11.0	77.6 ± 11.3	76.6 ± 11.1	<0.001
Grip, kg	30.0 ± 9.5	32.2 ± 9.8	30.2 ± 9.2	27.6 ± 8.8	<0.001
Hemoglobin, g/L	141.4 ± 15.3	138.9 ± 17.2	142.5 ± 14.2	142.8 ± 14.0	<0.001
Serum creatinine, μmol/L	70.8 ± 23.6	66.4 ± 15.1	70.0 ± 27.7	76.0 ± 24.8	<0.001
Fasting glucose, mmol/L	5.9 ± 1.6	5.3 ± 1.0	5.9 ± 1.7	6.4 ± 1.8	<0.001
Total cholesterol, mmol/L	5.5 ± 1.1	5.2 ± 1.0	5.7 ± 1.1	5.7 ± 1.2	<0.001
Triglycerides, mmol/L	1.6 ± 1.3	1.4 ± 1.3	1.7 ± 1.4	1.6 ± 1.0	<0.001
LDL cholesterol, mmol/L	3.2 ± 0.9	2.9 ± 0.8	3.3 ± 0.9	3.3 ± 1.0	<0.001
ODI, events/h	7.1 ± 7.8	6.3 ± 7.6	6.6 ± 7.4	8.4 ± 8.4	<0.001
OSA groups, no.(%)					<0.001
Normal	3068 (55.6)	1122 (63.4)	1106 (57.9)	840 (45.7)	
Mild	1714 (31.1)	440 (24.9)	595 (31.2)	679 (36.9)	
Moderate to severe	737 (13.4)	208 (11.8)	208 (10.9)	321 (17.4)	
Average oxygen saturation, (%)	96.5 ± 1.7	97.0 ± 1.7	96.5 ± 1.5	96.0 ± 1.7	<0.001
Minimum oxygen saturation, (%)	86.1 ± 5.8	86.6 ± 6.1	86.4 ± 5.3	85.4 ± 5.9	<0.001
CT 90, (%)	2.1 ± 7.6	1.9 ± 7.7	1.8 ± 6.7	2.6 ± 8.3	0.002
Poor sleep, no.(%)	2533 (53.2)	708 (46.6)	876 (52.8)	949 (59.9)	<0.001

Data are expressed as mean ± SD and number (%).

*Abbreviation*: *CT90% *Percentage of sleep time spent with SpO2<90%, *CVD *Cardiovascular diseases, *LDL *Low-density lipoprotein, *OSA *Obstructive sleep apnea, *ODI *Oxygen desaturation index

The original article [1] has been updated.
