# Supplementary figures and images for: Age-dependent association between obstructive sleep apnea and self-reported history of fractures: a community-based study
Source: BMC Public Health. 2025 Dec 23;25:4291. doi: 10.1186/s12889-025-25593-w (PMC12729138; doi:10.1186/s12889-025-25593-w)

**
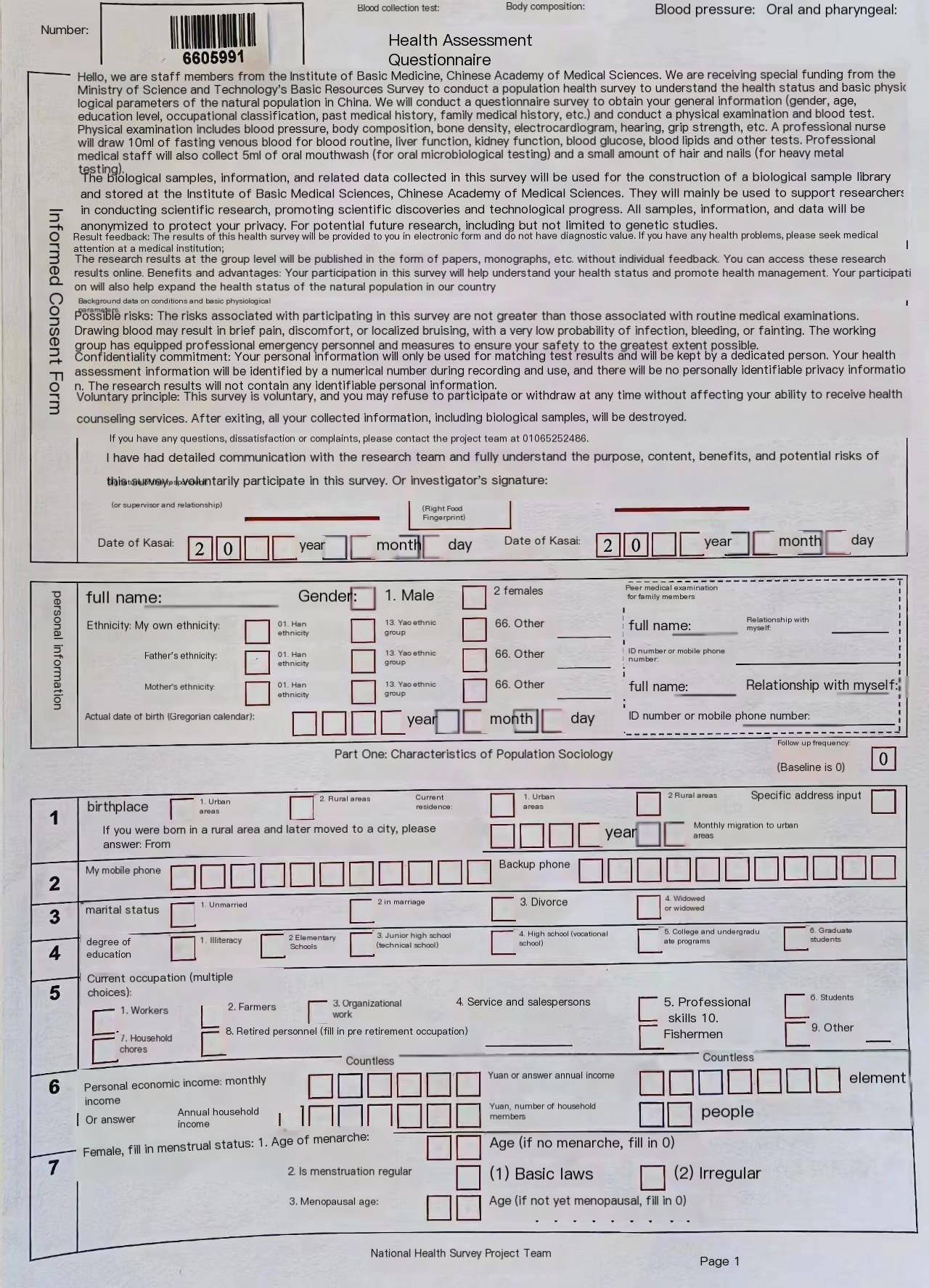
Supplementary data:** Questionnaires used in the study.


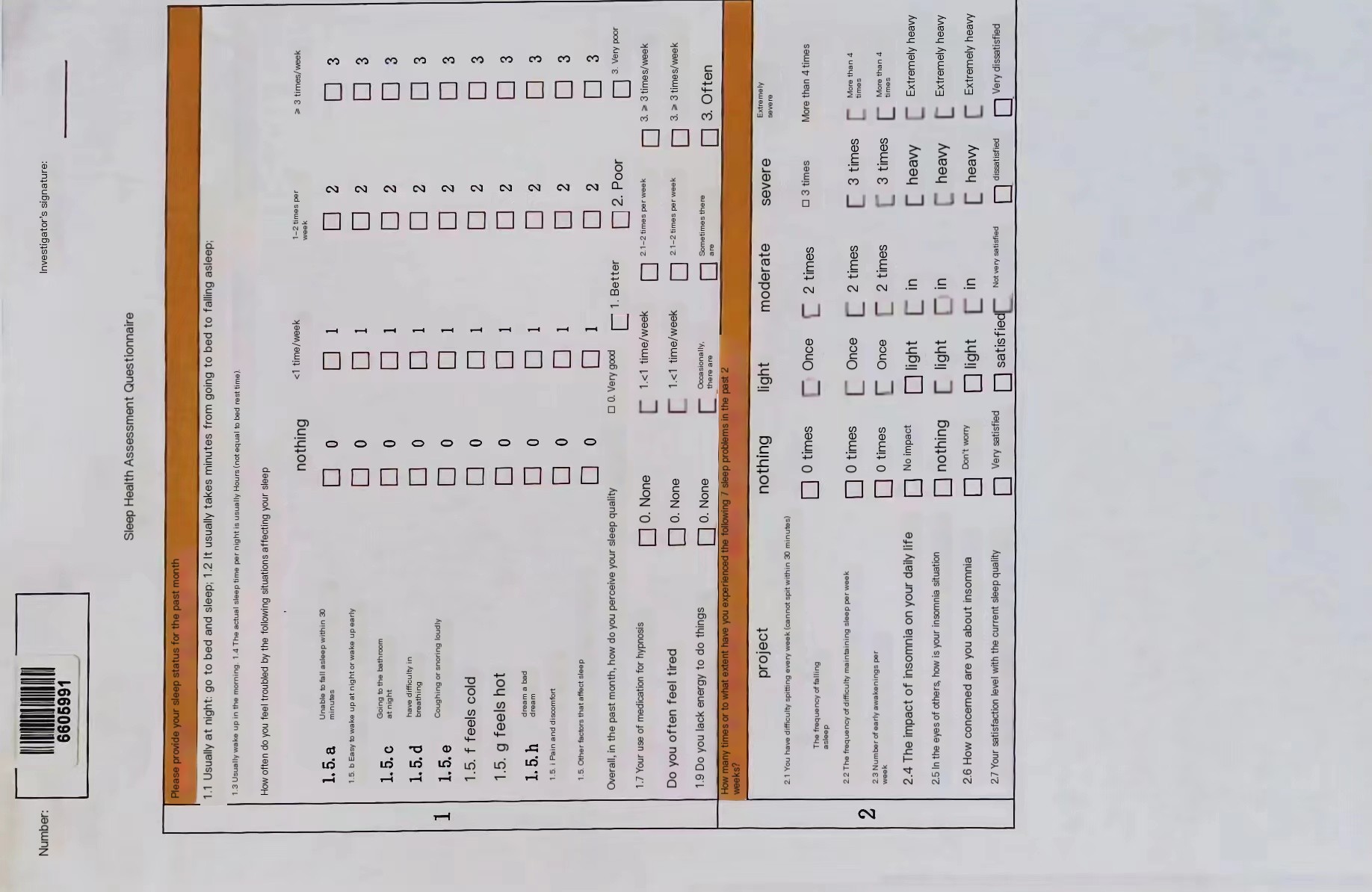


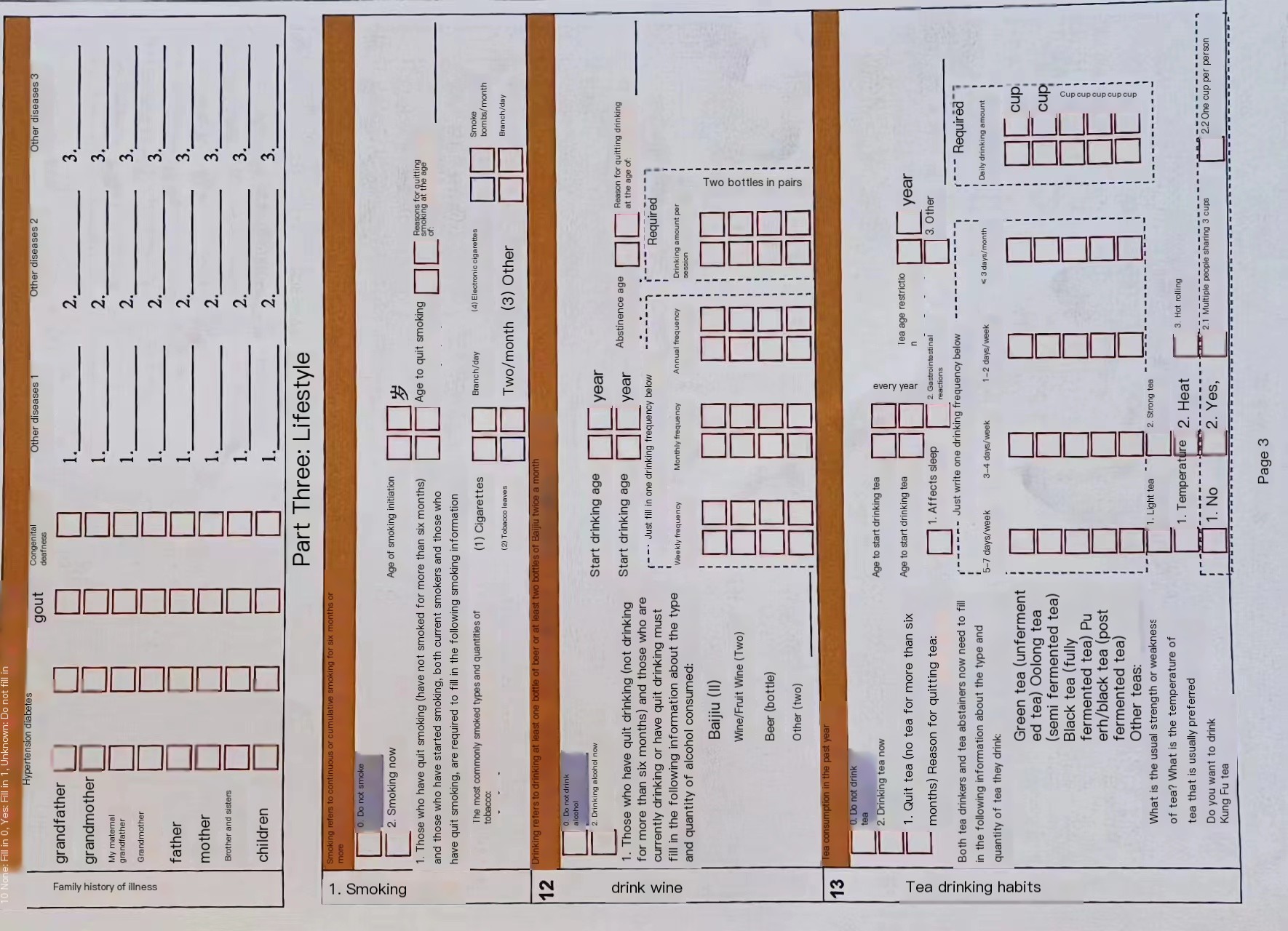


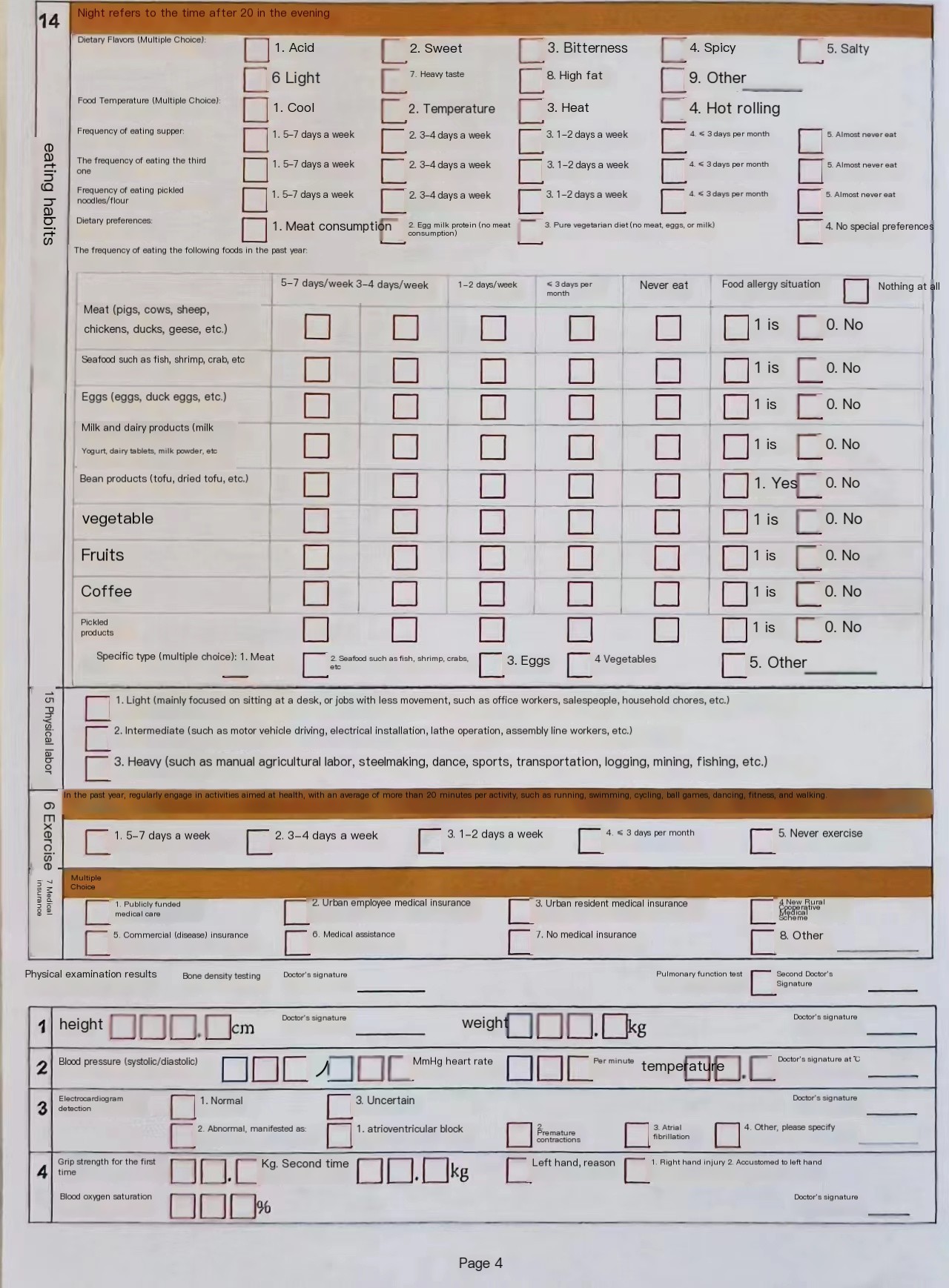

Supplement: Supplementary file 1 — Supplementary Material 1. [file 12889_2025_25593_MOESM1_ESM.docx]
