# Supplementary material for: Age-dependent association between obstructive sleep apnea and self-reported history of fractures: a community-based study
Source: BMC Public Health. 2025 Dec 23;25:4291. doi: 10.1186/s12889-025-25593-w (PMC12729138; doi:10.1186/s12889-025-25593-w)
Supplement: Supplementary file 2 — Supplementary Material 2. [file 12889_2025_25593_MOESM2_ESM.docx]

**Supplemental Online Content**

**Junzhi Chen, Guangliang Shan, Yaoda Hu et al.** **Age-dependent association between obstructive sleep apnea and self-reported history of fractures: A community-based study**

**e-Figure 1.** Flow diagram indicating derivation of final analyzed study sample

**e-Figure 2.** (a)show the weight of WISM, (b and c) show the wear location of the device on the palmar thenar major muscles

**
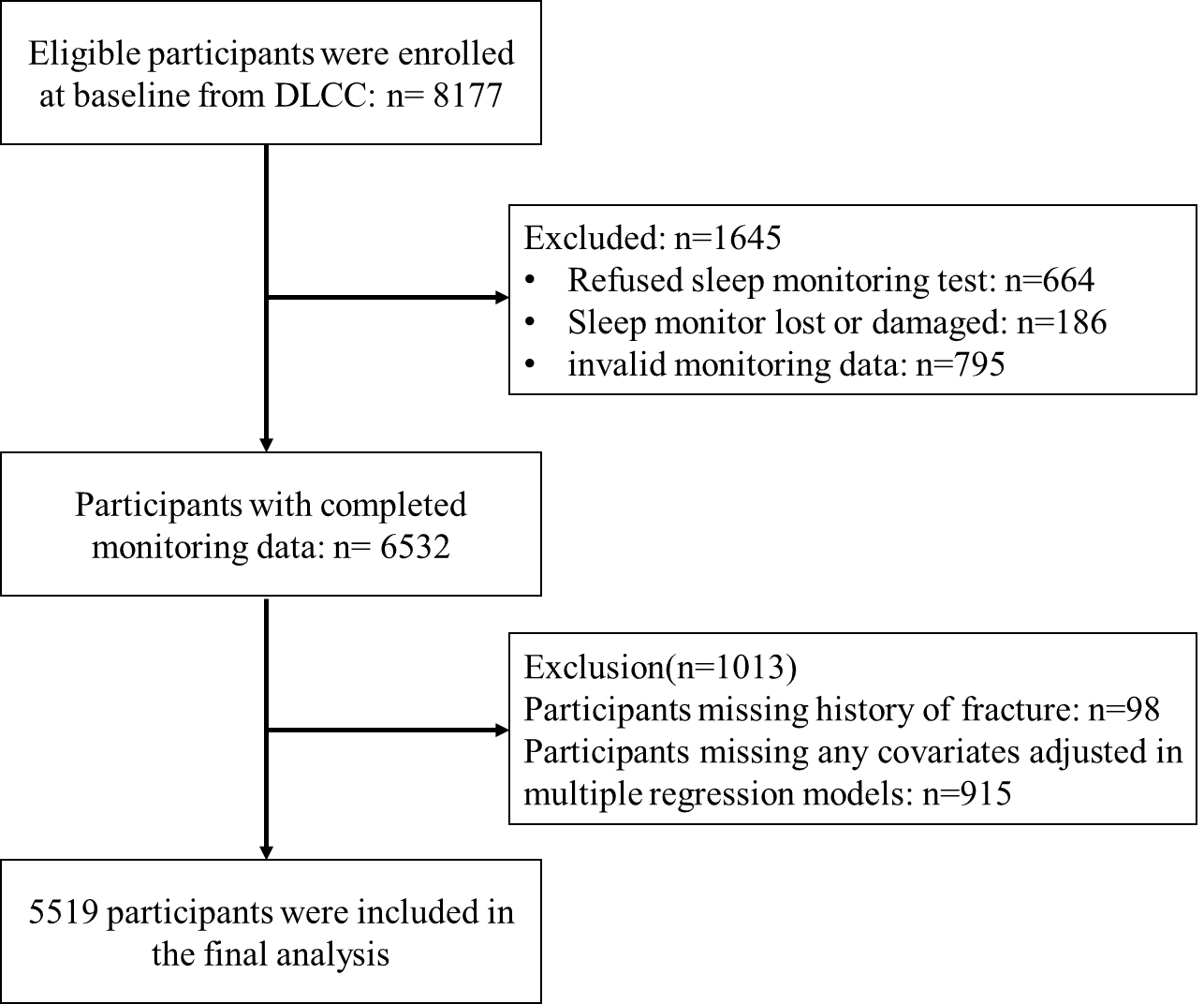
**

**e-Figure 1.** Flow diagram indicating derivation of final analyzed study sample


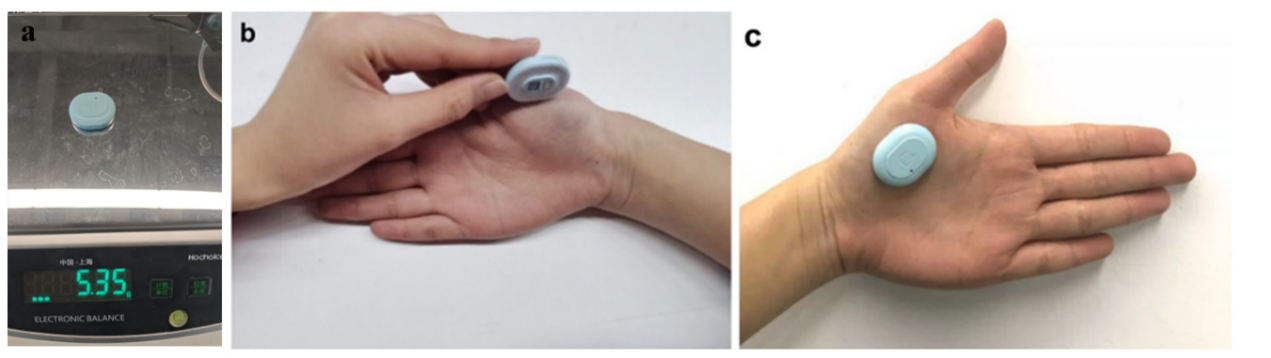


**e-Figure 2.** (a)show the weight of WISM, (b and c) show the wear location of the device on the palmar thenar major muscles
